# Rapid and sensitive detection of *Oncorhynchus mykiss* adulteration in *Salmo salar* by recombinase polymerase amplification combined with lateral flow strip: RPA-LFS assay for *Oncorhynchus mykiss* adulteration in *Salmo salar*

**DOI:** 10.3724/abbs.2022079

**Published:** 2022-07-12

**Authors:** Qiankun Yang, Xia Liu, Lei Wang, Guili Yu, Haitao Yang, Hang Fu, Xueqing Li, Nana He, Hong Yu, Jingquan Dong

**Affiliations:** 1 Jiangsu Key Laboratory of Marine Bioresources and Environment Co-Innovation Center of Jiangsu Marine Bio-industry Technology Jiangsu Key Laboratory of Marine Pharmaceutical Compound Screening Jiangsu Ocean University Lianyungang 222005 China; 2 Department of Laboratory Medicine the Second People’s Hospital of Lianyungang City Lianyungang 222000 China; 3 School of Biotechnology Jiangsu University of Science and Technology Zhenjiang 212018 China

*Oncorhynchus mykiss*, a kind of freshwater fish, belongs to the *Salmonidae* family and *Oncorhynchus* genus [1]. *O*. *mykiss* is farmed in more than 20 cities in China, being one of the most common cold-water farming fish. The wide range of *O*. *mykiss* may attribute to its rapid growth and tolerance of different environmental conditions. Salmo salar attracts customers’ interest for its delicious taste and high nutrient contents. However, wild *S*. *salar* supply is far less than high demands, and artificial breeding faces difficulties in yield and cost. External morphological characteristics can distinguish full-fledged fish, but it is difficult to distinguish after processing with pigments for the white and blue *O*. *mykiss* meat displaying orange color and texture [2]. The food industry provides cheaper *O*. *mykiss* adulteration in *S*. *salar*, infringing consumers’ rights and interests. Moreover, farmed *O*. *mykiss* in fresh water may risk parasite infection, and customers who eat raw food face food safety concerns [3].

Varieties of detection methods have been applied to food-authentication identification. Earlier techniques of liquid chromatography, electrophoretic profiling, and sensory analysis are facing problems of specificity and sensitivity [4]. DNA-based techniques surpass the limitations and become a primary identification trend in food industry for their excellent amplification ability [5]. However, the accumulated DNA amplification products need to be further identified by combination with the DNA sequencing (also known as DNA barcoding technology), agarose gel electrophoresis, or amplification curve and dissolution curve analysis (namely real-time PCR). This makes the amplification and detection highly dependent on the precision thermal cycling instrument and professional technicians. To overcome these limitations, isothermal amplification methods, such as loop-mediated isothermal amplification (LAMP), rolling circle amplification (RCA) and recombinase polymerase amplification (RPA) significantly reduce the dependence on thermal cycling instrument and can complete DNA amplification with an isothermal heater device [6]. Among them, RPA technology is far more promising for its similar to body heat temperature triggered reaction and short amplification time.

RPA amplifies DNA under a constant temperature. Briefly, the double strain DNA template is scanned by complexes formed by the UvsX and primer. Areas of homologous sequences are opened by UvsX, replaced by primer, and stabilized by gp32. The exposed 3′ end of the primer is accessible to Bsu polymerase for primer extension under body heat temperature ( Supplementary Figure S1A). With the aid of UvsY, UvsX is continuously bound and separated from the primer, making a dynamic cycle process mediated by ATP ( Supplementary Figure S1B). The whole process could be performed under a wide temperature ranging from 37°C to 45°C within 30 min. However, on-site RPA amplification for *O*. *mykiss* adulteration identification in *S*. *salar* has not been reported. To make on-site detection possible, RPA amplification is usually combined with lateral flow strips (LFS). In combination with chemicals-labeled primers, amplification products are modified with fluorescein isothiocyanate (FITC) and biotin at both ends. With mouse anti-FITC antibody functionalized gold nanoparticles (AuNPs) specifically capturing the labeled products, colored signals are observed visually. In detail, the RPA products migrate through the conjugate pad and react with anti-FITC AuNPs. Then, streptavidin coated on the test line will bind with AuNPs-conjugated RPA products, displaying positive signals. Those unconjugated anti-FITC antibody molecules will directly migrate to the control line coated with anti-mouse antibody and show red color verifying its validity ( Supplementary Figure S1C).

Here, we developed a rapid, visual, instrument-free method for on-site detection of *O*. *mykiss* adulteration in *S*. *salar*. The improved RPA-LFS assay could be performed using crude tissue lysate as templates and amplified under body-heat triggered reaction. The whole process could be accomplished within 40 min, from sample preparation to readable results. The data would provide new insight for on-site food adulteration identification.

To improve the accuracy of on-site inspection, the *myoglobin* ( *mb*) gene sequences of *O*. *mykiss* and *S*. *salar* were aligned, and primers and probe with high specificity were designed targeting the low homology areas using Primer Premier 3.0 software (Premier Biosoft International, San Francisco, USA). According to the principle of RPA primer design [7], the probe is designed in region 2 ( Supplementary Figure S2), and two pairs of primers were designed upstream and downstream of the probe ( Figure 1A). The DNAMAN software is used to analyze cross dimer ( Figure 1B). After screening and evaluation in theory, two pairs of primers and probe were verified to be suitable for RPA-LFS assay (named F1, F2, R1, R2, and P). Next, RPA-LFS assays were performed using the TwistAmp ^®^ DNA Amplification nfo Kit (TwistDx, Maidenhead, UK) according to the manufacturer’s instructions with a little modification referenced to our previous studies [8]. The results showed that a positive signal appeared at the *O*. *mykiss* group, while negative signals occurred at the *S*. *salar* and NTC groups when using primer and probe pairs of F1/R1/P ( Table 1). For primer and probe pairs of F1/R2/P, positive signal appeared at the *O*. *mykiss* group, and false positive signal appeared at the *S*. *salar* group, while negative signals occurred at the NTC groups. For primer and probe pairs of F2/R1/P and F2/R2/P, positive signals appeared at the *O*. *mykiss* group, while false positive signals occurred at the *S*. *salar* and NTC groups ( Figure 1C). Finally, primer and probe pairs of F1/R1/P could distinguish *O*. *mykiss* from *S*. *salar* without any false signal interference and be used for the developed RPA-LFS assay in detecting *O*. *mykiss* adulteration in *S*. *salar*.

**Figure 1:**
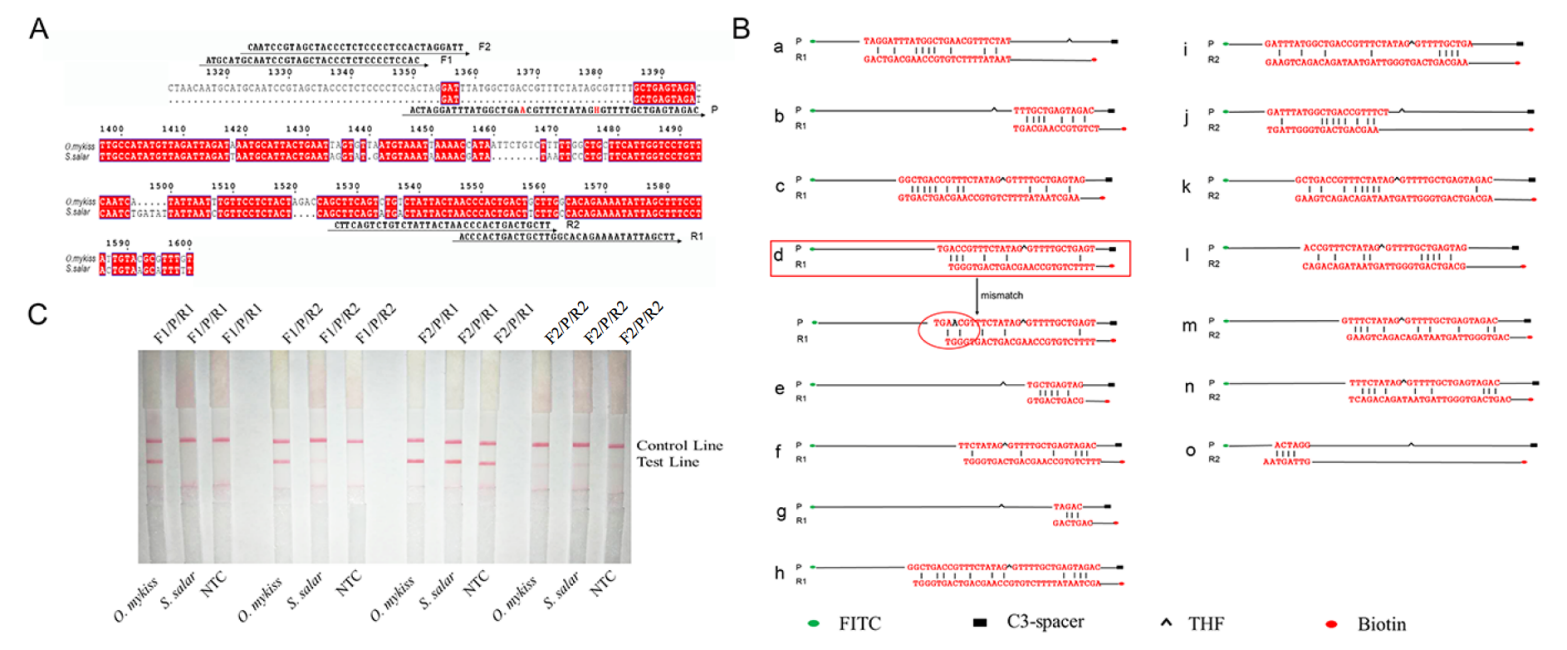
Figure 1 Designing and screening of primers and probes (A) Positions of primers and probes. The mb genes of S. salar and O. mykiss were compared, and the probes were designed to find regions with large differences (region 2). Primers were designed upstream and downstream of the probes with sequence name on the right. The arrow indicates the direction of the sequence, alongside the sequence name. (B) Cross dimer of analysis probe and reverse primer. The probe was analyzed with two reverse primers separately. The shapes and their representative labeling groups are listed at the bottom of the image. On the left are the names of primers and probes and the number of primer-dimer combinations. (C) Screening of primer-probe sets for RPA-LFS. The name of each combined primer and probe set is indicated on the top of the strip. NTC: no template control. The positions of test and control lines are marked on the right. Each image is the representative of three independent results.

**Table:** **Table 1** Sequences of primers and probe

Name	Sequence (5′→3′)
om-F1	ATGCATGCAATCCGTAGCTACCCTCTCCCCTCCAC
om-F2	CAATCCGTAGCTACCCTCTCCCCTCCACTAGGATT
om-R1	Biotin-AAGCTAATATTTTCTGTGCCAAGCAGTCAGTGGGT
om-R2	Biotin-AAGCAGTCAGTGGGTTAGTAATAGACAGACTGAAG
om-Probe	FITC-ACTAGGATTTATGGCTGAACGTTTCTATAG[THF]GTTTTGCTGAGTAGAC-/C3-spacer
Univ-F	CACGACGTTGTAAAACGACACYAAICAYAAAGAYATIGGCAC
Univ-R	GGATAACAATTTCACACAGGACITCAGGGTGWCCGAARAAYCARAA

The base labeled in red indicated mismatched base to avoid non-specific amplification.

Next, to explore the suitable temperature of RPA-LFS detection, the assay was conducted under different temperatures ranging from 30°C to 45°C for 30 min. As shown in Figure 2A, positive signals occurred at all the reaction time points, the red lines intensified along with increasing temperature, reached a peak at 42°C, and then declined. The optimized reaction temperature was between 37°C and 42°C. The traditional PCR-based methods need three stages of denaturation, annealing, and extension through temperature changes, necessitating the use of expensive thermal cycling equipment in the reaction process. However, the RPA-LFS method is close to body temperature. The template can be amplified without expensive heating equipment, which conforms to the on-site detection requirements

**Figure 2:**
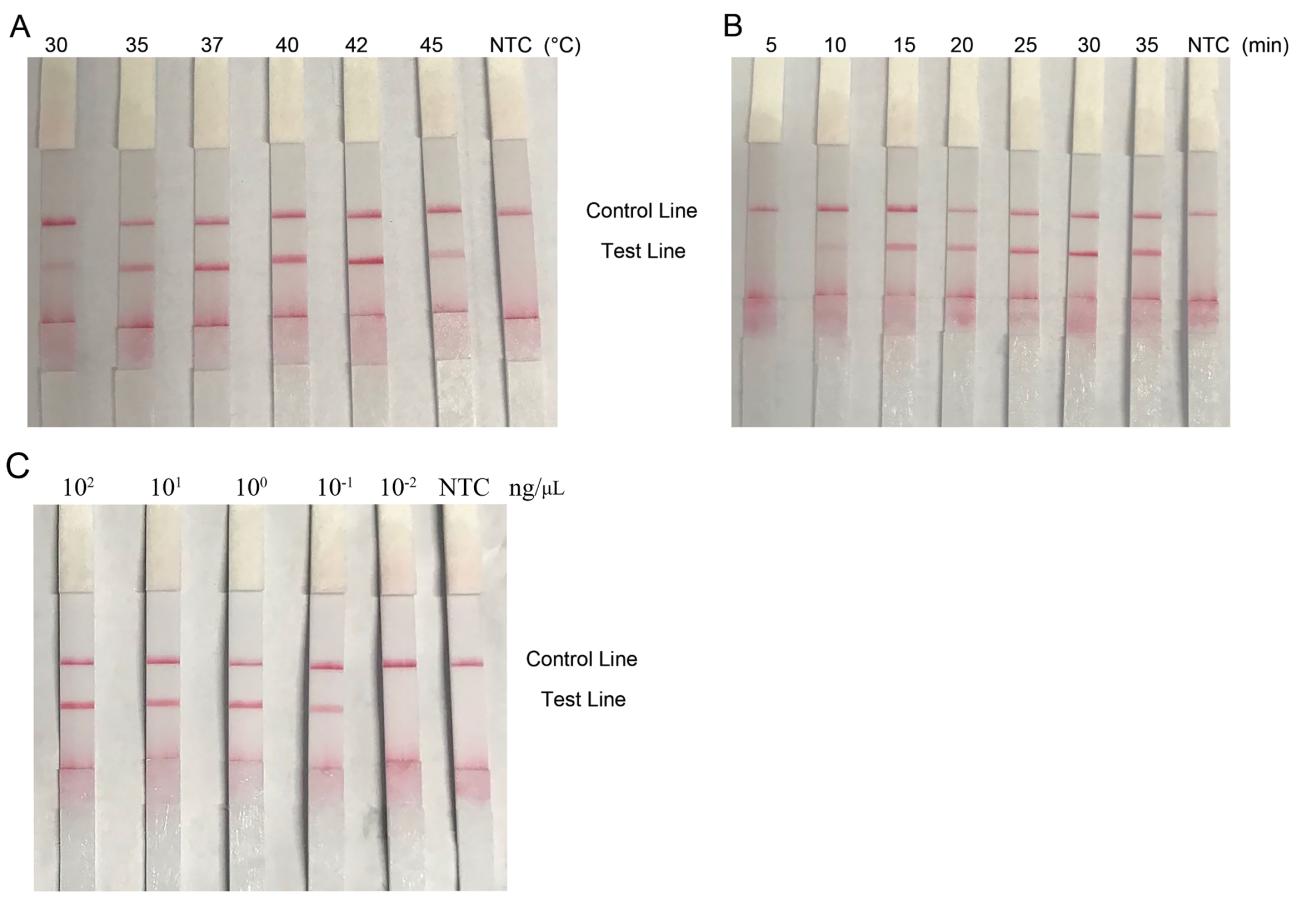
Figure 2 Determination of reaction temperature, time and reaction sensitivity of the RPA-LFS assay (A) Reaction temperature. (B) Reaction time. (C) Reaction sensitivity. The parameters of temperature, time and templates concentration are indicated on the strip top. NTC: no template control. The positions of test and control lines are marked on the right. Each image is the representative of three independent results.

To optimize the reaction time, the RPA-LFS assay was conducted for different time intervals ranging from 5 min to 35 min at 37°C. Results showed that no RPA products were found in the first 5 min, the red signals appeared at 10 min and intensified as reaction time increased, peaked at 30 min and then declined. The optimized reaction time was between 25 min and 30 min ( Figure 2B). It takes about 1~2 h for qPCR to detect *O*. *mykiss* from reaction to the presentation of results [2]. The detection of *O*. *mykiss* by PCR not only requires 1~2 h of reaction time, but also needs additional time to perform agarose gel electrophoresis, nucleic acid staining and UV detection to obtain the detection results. The reaction time of *O*. *mykiss* detection with LAMP is about one hour [9]. Overall, the detection time based on the RPA-LFS detection method is relatively short, including 10 min of RPA reaction process and 2 min of the detection process using LFS.

To make the RPA-LFS assay more suitable for on site detection, crude *O*. *mykiss* DNA were prepared. *O*. *mykiss* tissues (30 μg) were cut into thin slices, transferred to a centrifuge tube containing 600 μL of Trizol (Invitrogen, Carlsbad, USA), shaken by hands for 30 s, and lysed at room temperature for 1 min. Then, 350 μL of extraction buffer (phenol:chloroform:isoamyl alcohol=25:24:1) and 30 μL of nanometer magnetic beads (NANOEAST, Nanjing, China) were added into the tube, shaken by hands violently for 10 s, and then placed on the magnet. The supernatants were discarded, and the beads were washed twice with 75% ethanol. DNA was released by elution with 60 μL of ddH _2_O. The prepared crude *O*. *mykiss* DNA samples were diluted in 10-fold gradient and used in the detection sensitivity assay. The results showed that the detection limit of RPA-LFS was 0.1 ng/μL, indicating that the established RPA-LFS assay for *O*. *mykiss* identification exhibited high sensitivity and high tolerance for sample purity ( Figure 2C).

To further explore the feasibility of the on-site body-heat triggered RPA-LFS method, a total of 36 volunteers took part in the authentication of *S*. *salar*. Firstly, crude DNA was prepared and provided for each volunteer to make RPA mixture. Then, they all heat the reaction tubes by hand for 30 min and read the detection results with naked-eyes ( Supplementary Figure S3). Results showed that there were 7 *O*. *mykiss* positive samples and 26 *S*. *salar* positive samples. At the same time, DNA barcoding detection was carried out and yielded consistent results ( Supplementary Table S1)

At present, *O*. *mykiss* as an adulterated object in the *S*. *salar* may carry parasites and the food safety of consumers is facing challenges [3]. RPA-LFS has been used to identify *S*. *salar* recently [10], however, the positive detection signals could only determine the cooked food containing *S*. *salar* but not *O*. *mykiss* adulteration. Instead, development of RPA-LFS assay for *O*. *mykiss* will recognize any mixture of *S*. *salar* and *O*. *mykiss* or *O*. *mykiss* alone, which usually occur in China’s food industries. Meanwhile, the improved RPA-LFS assay for *O*. *mykiss* sharply simplifies the sample preparation procedures and amplification reaction condition, which makes the *O*. *mykiss* adulteration identification more suitable for on-site detection.

In conclusion, a novel method for on-site detection of *O*. *mykiss* authentication in *S*. *salar* was established based on RPA-LFS technology. The body temperature replaced the conventional sophisticated temperature control equipment to finish DNA amplification. The developed RPA-LFS system allows the detection to be completed within 40 min under temperature of 37°C–42°C. The proposed method is not only designed for on-site screening of *O*. *mykiss* authentication in *S*. *salar* but also applicable to detection of adulteration of other foods, revealing its great potential for food safety analysis.

## Supporting information

22059Table

## Supplementary Data

Supplementary data is available at *Acta Biochimica et Biophysica Sinica* online.
