# Supplementary material for: Rapid and sensitive detection of Oncorhynchus mykiss adulteration in Salmo salar by recombinase polymerase amplification combined with lateral flow strip : RPA-LFS assay for Oncorhynchus mykiss adulteration in Salmo salar
Source: Acta Biochim Biophys Sin (Shanghai). 2022 Jul 12;54(7):1039–42. doi: 10.3724/abbs.2022079 (PMC9828328; doi:10.3724/abbs.2022079)
Supplement: 22059Table [file 22059Table.pdf]

Table 1. Sequence of primers and probe

Name	Sequence (5′→3′)
om- F1	ATGCATGCAATCCGTAGCTACCCTCTCCCCTCCAC
om- F2	CAATCCGTAGCTACCCTCTCCCCTCCACTAGGATT
om-R1	Biotin-AAGCTAATATTTTCTGTGCCAAGCAGTCAGTGGGT
om-R2	Biotin- AAGCAGTCAGTGGGTTAGTAATAGACAGACTGAAG
om-Probe	FITC-ACTAGGATTTATGGCTGAACGTTTCTATAG[THF]GTTTTGCTGA GTAGAC-/C3-spacer/
Univ-F	CACGACGTTGTAAAACGACACYAAICAYAAAGAYATIGGCAC
Univ-R	GGATAACAATTTCACACAGGACITCAGGGTGWCCGAARAAYCARAA

The base labeled in red indicated mismatched base to avoid non-specific amplification.
